# 

**Published:** 1908-03

**Authors:** 


### CONTENTS.

ORIGINAL COMMUNICATIONS—

The Diagnosis and Treatment of Penetrating Wounds of the Abdomen. (Abstract.) By Floyd W. McRae. Atlanta, Ga. .. .. .. .. .. .. .. .. .. .. .. .. .. .. .. ..709

Adehesive Plaster for the Cure of Certain External Diseases and Lesions. By M. B. Huthins, M. D., Atlanta, Ga. Chief of Skin and Cancer Service. Presbyterian Hospital, Baptist Tabernacle Infirmary and Wesley Memorial Hospital, etc. .. .. .. .. .. .. ..717

The Diagnosis of Incipient Pathological Changes in the Kidney by Means of Renal Epithelium. By A. T. Gaillard, M. D., Atlanta.. .. .. .. .. .. .. .. .. .. .. .. ..725

Malarial Hemoglobinuria. By E. F. Jones. M. D., Midville, Ga. .. .. .. .. .. .. .. ..741

CORRESPONDENCE. .. .. .. .. .. .. .. .. .. .. .. .. .. .. .. .. .. .. .. .. ..746

EDITORIAL NOTES AND COMMENTS—

Increased Editorial Staff. .. .. .. .. .. .. .. .. .. .. .. .. .. .. .. .. 747

Grady Hospital Affair Finally Settled. .. .. .. .. .. .. .. .. .. .. .. .. ..748

How to Burn Coal Without an Excess of Smoke. .. .. .. .. .. .. .. .. .. .. ..749

Juvenile Protective Association. .. .. .. .. .. .. .. .. .. .. .. .. .. .. ..750

Medical Legislation and Enforcement of Present Laws Against Illegal Practitioners.. .. ..752

NEWS AND NOTES. .. .. .. .. .. .. .. .. .. .. .. .. .. .. .. .. .. .. ..753 to 759

BOOK REVIEWS. .. .. .. .. .. .. .. .. .. .. .. .. .. .. .. .. .. .. .. ..760-761

SELECTIONS AND ABSTRACTS. .. .. .. .. .. .. .. .. .. .. .. .. .. .. ..762 to 764

